# Regulatory Effects of 198-bp Structural Variants in the *GSTA2* Promoter Region on Adipogenesis in Chickens

**DOI:** 10.3390/ijms25137155

**Published:** 2024-06-28

**Authors:** Wangyu Li, Meng Xu, Zihao Zhang, Jiaying Liang, Rong Fu, Wujian Lin, Wen Luo, Xiquan Zhang, Tuanhui Ren

**Affiliations:** 1 Department of Animal Genetics, Breeding and Reproduction, College of Animal Science, South China Agricultural University, Guangzhou 510642, Chinafurong325@163.com (R.F.); linwujian0915@163.com (W.L.); luowen729@scau.edu.cn (W.L.); 2 Guangdong Key Laboratory of Genome and Molecular Breeding of Agricultural Animals and Key Laboratory of Chicken Genetic Breeding and Reproduction, Ministry of Agriculture, Guangzhou 510642, China; 3 State Key Laboratory of Swine and Poultry Breeding Industry, Guangzhou 510642, China; 4 College of Veterinary Medicine, Jilin University, Changchun 130062, China; 5 College of Coastal Agricultural Sciences, Guangdong Ocean University, Zhanjiang 524088, China; jayhozhang@163.com

**Keywords:** structural variation, adipogenesis, preadipocyte proliferation and differentiation

## Abstract

Molecular breeding accelerates animal breeding and improves efficiency by utilizing genetic mutations. Structural variations (SVs), a significant source of genetic mutations, have a greater impact on phenotypic variation than SNPs. Understanding SV functional mechanisms and obtaining precise information are crucial for molecular breeding. In this study, association analysis revealed significant correlations between 198-bp SVs in the *GSTA2* promoter region and abdominal fat weight, intramuscular fat content, and subcutaneous fat thickness in chickens. High expression of *GSTA2* in adipose tissue was positively correlated with the abdominal fat percentage, and different genotypes of *GSTA2* exhibited varied expression patterns in the liver. The 198-bp SVs regulate *GSTA2* expression by binding to different transcription factors. Overexpression of *GSTA2* promoted preadipocyte proliferation and differentiation, while interference had the opposite effect. Mechanistically, the 198-bp fragment contains binding sites for transcription factors such as C/EBPα that regulate *GSTA2* expression and fat synthesis. These SVs are significantly associated with chicken fat traits, positively influencing preadipocyte development by regulating cell proliferation and differentiation. Our work provides compelling evidence for the use of 198-bp SVs in the *GSTA2* promoter region as molecular markers for poultry breeding and offers new insights into the pivotal role of the *GSTA2* gene in fat generation.

## 1. Introduction

Chickens are the most common farm animals worldwide and are known for their strong reproductive ability, short growth cycle, and high feed conversion efficiency. They can produce nutritionally rich eggs and meat products at a low cost within a relatively short production period, making them an ideal source of animal protein for humans [1]. Over the past few decades, genetic improvements have greatly enhanced the growth performance of broiler chickens [2]. However, the accelerated growth of modern broilers has given rise to a range of developmental and metabolic disorders [3,4]. The excessive accumulation of abdominal fat represents a prevalent concern in poultry production, as it adversely impacts feed efficiency and carcass yield and imposes substantial economic losses due to waste processing [5].

Glutathione S-transferases (GSTs) are a ubiquitous group of intracellular enzymes that facilitate the conjugation of glutathione with diverse exogenous and endogenous substances [6]. During adipogenesis in 3T3-L1 cells, there is a decrease in the ratio of glutathione to oxidative glutathione, shifting the redox state toward oxidation and accelerating fat formation [7]. GSTs can be categorized into three distinct gene families, namely, mitochondrial GSTs and cytosolic GSTs. Research has indicated the vital involvement of mitochondrial GSTs in obesity, diabetes, and lipid metabolism [8], while cytosolic GSTs can be divided into seven types in mammals, including α, μ, π, σ, θ, ζ, and ω, which are highly conserved among species and share common evolutionary pathways [6,9]. Among them, glutathione S-transferase alpha 2 (*GSTA2*) exhibits pronounced expression in both human liver and breast tissue [10]. *GSTA2*, a member of the GST gene family, has a protective effect against oxidative stress. Overexpression of *GSTA2* compensates for increased oxidative stress, while downregulation of *GSTA2* impairs this effect [11,12]. Previous studies have shown the involvement of *GSTA2* in carotenoid pigmentation in pheasants and identified *GSTA2* as a candidate gene for carotenoid deposition in Botia dario fish [13,14]. However, the functional mechanism underlying chicken *GSTA2* activity remains unclear and warrants further investigation.

Structural variations (SVs) are large-scale variants in the genome with lengths greater than 50 base pairs (also known as long sequence indels) and can include deletions, insertions, duplications, and inversions, representing the most extensive alterations in the human genome [15,16]. Genome rearrangements generate a multitude of SVs, which are predominantly found in the noncoding regions of genes. These SVs can have substantial effects on mRNA splicing and processing, transcriptional regulatory elements, genome folding and spatial organization, and even protein translation [17,18]. SVs represent a significant force of mutation capable of influencing genome evolution and function [19,20]. Compared to single-nucleotide polymorphisms (SNPs), SVs can explain a greater proportion of phenotypic variation [21]. Therefore, using SVs as markers for the direct analysis of genetic diversity holds promise for identifying causal loci influencing phenotypes. Increasing attention has been given to the important impact of SVs on animal phenotypes and human diseases. Many studies have demonstrated a close association between SVs and diseases such as cognitive disorders, obesity, and cancer in humans [15,19]. In studies of domesticated animals, the involvement of SVs has been noted in the regulation of the proliferation and differentiation of muscle cells in Boer goats. This effect is mediated by the dosage impact of overlapping genes, which potentially facilitates skeletal muscle development [22]. Researchers constructed a high-quality SV map for pigs and, in combination with genome-wide association analysis, found that SVs were primarily associated with skeletal size. It is speculated that different SVs may be one of the reasons for the variation in body size between commercial pigs and Chinese indigenous pigs [23].

Promoters are specialized DNA sequences capable of identifying and binding transcription factors, thereby initiating the process of transcription [24]. Promoters typically refer to regions within the upstream genomic sequence of the transcription start site (TSS), although sequences downstream of the TSS can also influence the initiation of gene transcription [25]. Earlier research identified two indels within the glutaminyl peptide cyclotransferase-like (*QPCTL*) promoter region that exhibit significant associations with body weight in chickens. These indels exert regulatory control over the proliferation and differentiation of muscle cells by influencing the expression of *QPCTL*, potentially influencing chicken growth [26,27]. An indel involving multiple alleles in the *CDKN3* promoter region is strongly correlated with economic traits in chickens, making it a promising molecular marker for chicken breeding [24]. Furthermore, an indel within the paired box 7 (*PAX7*) promoter region may have a significant impact on growth traits in cattle [28].

This study aimed to examine the impact of a 198-bp SV within the chicken *GSTA2* promoter region on chicken adipose traits, *GSTA2* expression, and the regulation of proliferation and differentiation in preadipocyte cells. The correlation between the 198-bp SVs of *GSTA2* and meat quality and carcass traits in chickens was analyzed. The tissue expression profile of *GSTA2* and the changes in *GSTA2* expression in the adipose tissues of chickens with high and low abdominal fat contents were investigated. Additionally, the changes in the expression of different *GSTA2* genotypes in the liver were analyzed. Furthermore, a dual-luciferase reporter assay was used to investigate the impact of transcription factors in the inserted fragment on *GSTA2* transcriptional activity. To investigate the role of *GSTA2* in immortalized chicken preadipocyte 1 (ICP1) cells, *GSTA2* overexpression and interference experiments were performed. Our findings revealed a notable correlation between the 198-bp SVs of *GSTA2* and various traits in chickens, including abdominal fat weight, subcutaneous fat thickness, and other traits. Moreover, the expression of *GSTA2* in adipose tissues was significantly positively correlated with the percentage of abdominal fat. Specifically, the 198-bp SVs regulated the gene expression of *GSTA2* by binding to different transcription factors, thereby modulating the proliferation and differentiation of ICP1 cells. The current study provides valuable insights into molecular breeding in chickens and sheds new light on the mechanisms underlying excessive fat deposition in chickens.

## 2. Results

### 2.1. Conservation and Protein Interactions of the GSTA2 Gene

**Figure 1:**
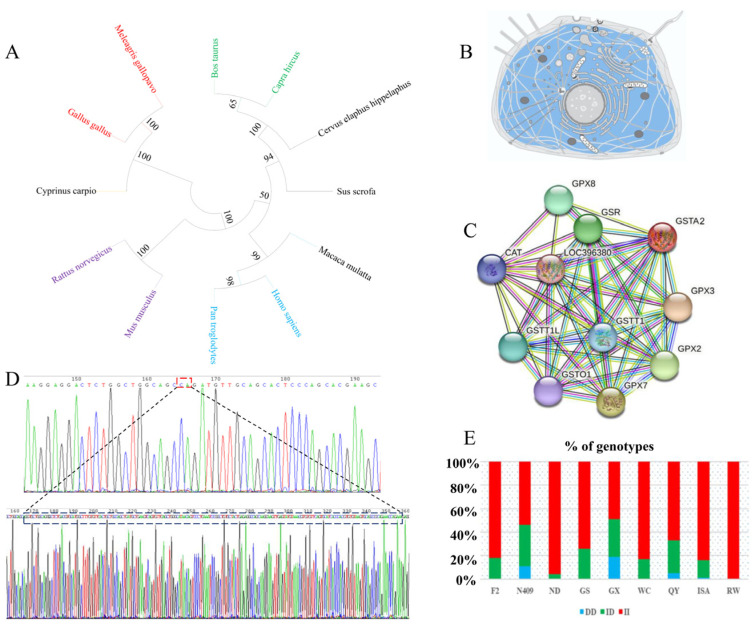
Bioinformatics analysis and gene genotyping of *GSTA2*. (**A**) Phylogenetic analysis of the *GSTA2* gene in different species. *Gallus gallus* (chicken), *Bos taurus* (cattle), *Capra hircus* (goat), *Sus scrofa* (pig), *Homo sapiens* (human), *Mus musculus* (house mouse), *Rattus norvegicus* (brown rat), *Meleagris gallopavo* (turkey), *Cervus elaphus* (red deer), *Pan troglodytes* (chimpanzee), *Macaca mulatta* (rhesus monkey), and *Cyprinus carpio* (carp). (**B**) Subcellular localization prediction of the *GSTA2* gene. (**C**) Protein–protein interaction prediction of the *GSTA2* gene. The purple lines represent experimentally validated interactions between proteins, the black lines represent coexpression relationships between proteins, the yellow lines represent text mining-based associations, and the blue lines represent gene neighborhood associations. (**D**) DNA sequencing files of the 198-bp SV of the *GSTA2* gene. (**E**) Percentages of the deletion/deletion (DD) (blue), insertion/deletion (ID) (green) and insertion/insertion (II) (red) genotypes in the different populations.

By aligning the amino acid sequences of *GSTA2* from various species, a phylogenetic tree was generated that revealed four distinct branches, each representing *GSTA2* from different species. *Gallus gallus*, *Meleagris gallopavo*, and *Cyprinus carpio* clustered together on the same major branch, indicating a close evolutionary relationship with a comparatively small genetic distance between them. *Homo sapiens*, *Macaca mulatta*, and *Pan troglodytes* clustered on other major branches (Figure 1A). *GSTA2* may be homologous among the species with smaller genetic distances.

Using the UniProt database, subcellular localization prediction was performed to determine the cellular localization of *GSTA2*. *GSTA2* is likely localized predominantly to the cytoplasm (Figure 1B). Additionally, the STRING database was used to predict potential interacting proteins in chicken GSTA2. The results showed that GSTA2 was potentially correlated with 10 different proteins, including GPX2, GPX3, GPX7, GPX8, GSTO1, and GSR (Figure 1C). These findings suggest that GSTA2 is sequence-conserved and interacts with multiple proteins in vertebrates.

### 2.2. Identification and Genotyping of the 198-bp SV

In this study, a 198-bp SV located in the promoter region of *GSTA2* was identified, as confirmed by DNA sequencing (Figure 1D and Figure S1). The PCR products were visualized through 1.5% gel electrophoresis, which revealed three genotypes: the 542-bp II genotype, the ID genotype (542-bp and 344-bp), and the 344-bp DD genotype (Figure S2).

### 2.3. Genetic Diversity of the 198-bp SV

To investigate the distribution pattern of the different genotypes among the nine populations, allele and genotype frequencies and genetic parameters were analyzed in a total of 1205 chickens from the nine different populations. The genotype frequency results showed that the DD genotype had the lowest frequency, and the II genotype had the highest frequency among all populations. In F2, ND, GS, WC and RW chickens, the DD genotype was not detected. Notably, only the II genotype was present in the RW chicken breeds (Figure 1E). These findings suggest that artificial breeding could influence the frequency of distinct *GSTA2* genotypes.

In all populations, the frequency of allele *I* was consistently greater than that of allele *D*. Notably, allele *I* exhibited the lowest frequency in GX and N409, while RW showed the highest frequency of allele *I*. The χ^2^ test results indicated that, except for N409, GX, and RW, the genotypic and allele frequencies of the 198-bp SV were in HWE across all populations (*p* > 0.05). The values of Ho, He, and Ne ranged from 0 to 0.35, 0 to 0.45, and 1 to 1.83, respectively. The PIC values ranged from 0.0 to 0.35, with GX, QY, and N409 displaying moderate polymorphism (0.25 < PIC < 0.50), while the remaining populations exhibited low polymorphism (PIC < 0.25) (Table 1).

### 2.4. Differential Selection of the 198-bp SV

To evaluate the potential occurrence of selective differentiation in the 198-bp SV of the *GSTA2* gene during domestication, the pairwise fixation index (Fst) was utilized as a measure of genetic differentiation between populations. The findings revealed a significant level of genetic differentiation between the ND and GX chickens, as well as between the GX and RW populations (0.15 < Fst < 0.25). There was a moderate level of genetic differentiation between the F2 and N409, GX, QY, ND and GX, QY, GX and WC, and GS and RW populations (0.05 < Fst < 0.15). Furthermore, we observed low genetic differentiation between almost all other population pairs (Fst < 0.05) (Table S2).

### 2.5. Association of the 198-bp SV with Chicken Carcass and Meat Traits

A mixed model was employed to examine the relationships between genotypes and economic traits. The results showed that the 198-bp SV of the two genotypes was significantly correlated with abdominal fat weight (AFW) (*p* < 0.05), subcutaneous fat thickness (SFT) (*p* < 0.05) and the content of chicken leg muscle crude fat (LMCF) (*p* < 0.01) (Table 2). Significantly, the II genotype exhibited higher values than did the ID genotype across all the associated traits.

### 2.6. Expression of GSTA2 Gene

**Figure 2:**
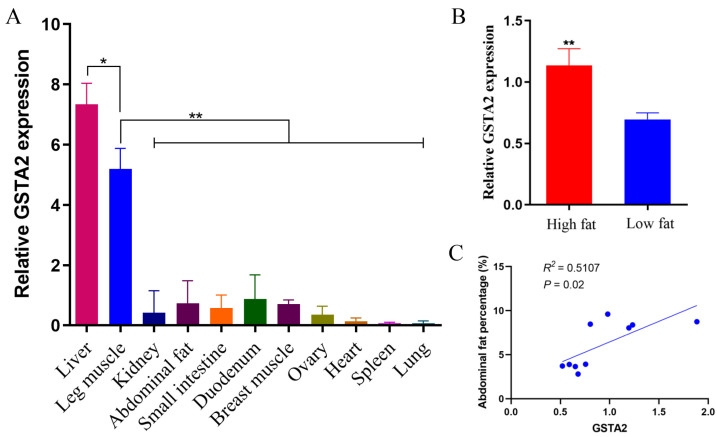
Relative expression of the *GSTA2* gene. (**A**) Relative expression levels of *GSTA2* in different tissues. (**B**) Expression levels of *GSTA2* in the abdominal fat of chickens with different abdominal fat contents. (**C**) Correlation between the expression of *GSTA2* in chicken abdominal fat and the percentage of abdominal fat. * *p* < 0.05; ** *p* < 0.01.

qPCR was utilized to measure the expression of *GSTA2* in 11 tissues of QY spotted-brown chickens, and *GSTA2* was found to be expressed in all tissues. Additionally, the liver and leg muscle tissues of chickens demonstrated significantly elevated expression levels of *GSTA2* compared to the remaining tissues (*p* < 0.01). Conversely, the heart, spleen, and lung tissues exhibited relatively lower expression levels (Figure 2A).

In addition, we investigated the expression of *GSTA2* in the adipose tissue of chickens categorized as having high or low abdominal fat. *GSTA2* expression was significantly greater in the high abdominal fat group than in the low abdominal fat group (*p* < 0.01) (Figure 2B). Additionally, a strong positive correlation was found between *GSTA2* expression and the abdominal fat percentage in chickens (*p* < 0.05) (Figure 2C).

### 2.7. Relative Expression of GSTA2 in Different Genotypes

The expression profile analysis revealed elevated levels of *GSTA2* expression in the liver tissues of chickens. Subsequently, we investigated the expression levels of *GSTA2* in liver tissues across different genotypes. Notably, the expression of the II genotype was markedly greater than that of the DD genotype (*p* < 0.05). Nevertheless, there was no significant difference between the II and ID genotypes.

**Figure 3:**
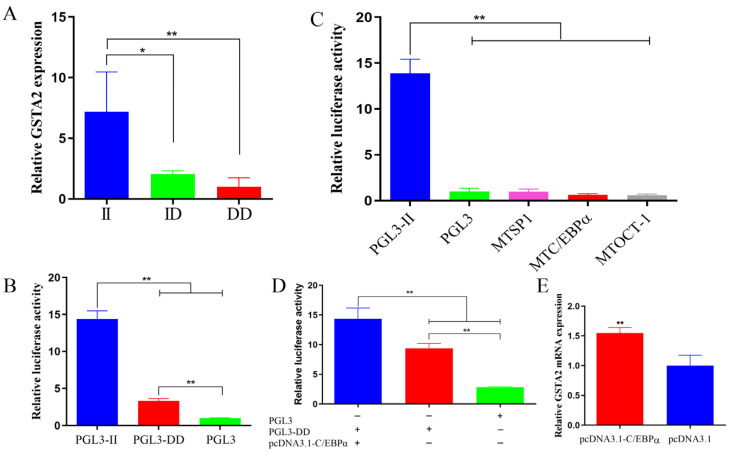
Transcription factors in the 198-bp SV of the *GSTA2* gene. (**A**) Expression levels of the *GSTA2* gene in the livers of the different genotypes. (**B**) Luciferase activity levels of the II and DD homozygous genotypes. (**C**) Luciferase activity levels after mutation of the transcription factor-binding sites. (**D**) Luciferase activity levels after cotransfection of the DD genotype luciferase and C/EBPα overexpression vectors. (**E**) Expression of *GSTA2* after overexpression of *C/EBPα* in ICP1 cells. * *p* < 0.05; ** *p* < 0.01.

Interestingly, the expression of the ID genotype exhibited an increasing trend compared to that of the DD genotype (Figure 3A). These findings highlight the influence of different genotypes on the expression of *GSTA2*.

### 2.8. Promoter Activity of GSTA2

The impact of SVs in different alleles on promoter activity was assessed using a dual-luciferase reporter assay. The results revealed a notable increase in luciferase activity for the pGL3-II vector compared to both the pGL3-DD and pGL3 vectors (*p* < 0.01). Additionally, the luciferase activity of the pGL3-DD vector was markedly greater than that of the pGL3 vector (*p* < 0.01) (Figure 3B). These results strongly suggest that SVs present in different alleles exert a notable influence on promoter activity.

### 2.9. Prediction and Validation of Transcription Factors in the 198-bp SV

The 198-bp SV of the *GSTA2* promoter was subjected to analysis for transcription factor-binding sites (TFBSs) using online software. The results revealed a total of 25 TFBSs within the insertion fragment of the *I* allele, encompassing 3 C/EBPα, 4 SP1, 1 OCT-1, and some other sites (refer to Figure S3). These findings indicate that the binding of specific transcription factors to the *I* allele has the potential to influence promoter activity.

To determine whether the binding of the potential transcription factor and the 198-bp insertion sequence affect the transcription activity of genes, three potential TFBSs for the transcription factors C/EBPα, SP1, and OCT-1 of the II genotype were mutated. The transcriptional activity of the target genes was then detected in DF-1 cells. The results suggested that after the mutation of these transcription factor-binding sites, the transcriptional activity of genes was markedly reduced (*p* < 0.01) (Figure 3C). These findings show that the 198-bp insertion sequence for the transcription factors C/EBPα, SP1 and OCT-1 can affect the transcriptional activity of *GSTA2*.

To further verify the impact of the C/EBPα transcription factor on *GSTA2* gene expression, pGL3-DD and *C/EBPα* overexpression vectors were cotransfected, and changes in the dual-luciferase activity were detected. The dual-luciferase activity in the cotransfection group with the overexpression vector and PGL3-DD was markedly greater than that in the PGL3-DD and PGL3 control groups (*p* < 0.01) (Figure 3D). Next, after *C/EBPα* was overexpressed in ICP1 cells, the changes in the expression of the *GSTA2* gene were tested. The findings showed that overexpression of C/EBPα markedly increased the expression of the *GSTA2* gene (*p* < 0.01) (Figure 3E). These findings provide additional evidence supporting the notion that C/EBPα has the potential to function as a transcription factor for *GSTA2*.

### 2.10. GSTA2 Promotes the Proliferation of ICP1

**Figure 4:**
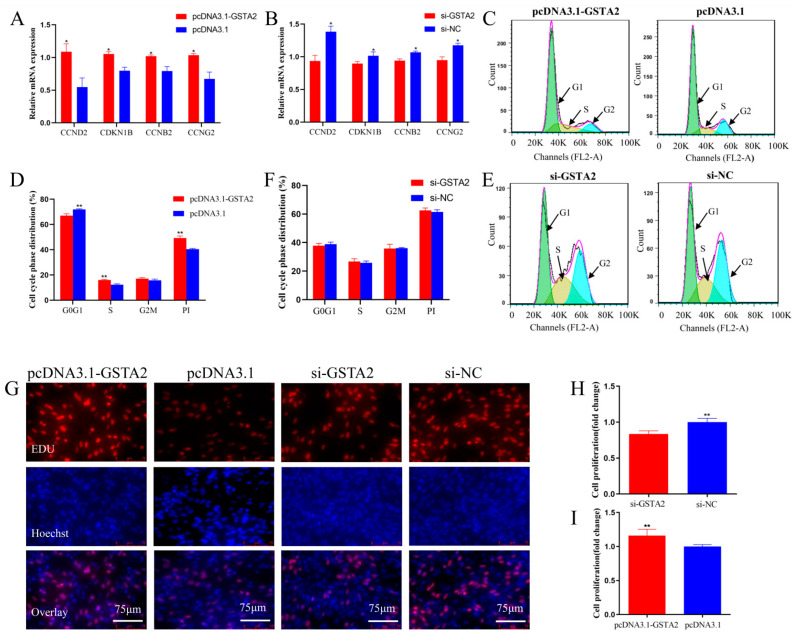
Promotion of chicken preadipocyte proliferation by the *GSTA2* gene. (**A**,**B**) Relative mRNA expression levels of proliferation-related genes were detected by qPCR after transfection of chicken ICP1 cells with the *GSTA2* overexpression plasmid and *GSTA2* siRNA for 48 h. (**C**–**F**) Cell cycle analysis after transfection of a *GSTA2* overexpression plasmid and *GSTA2* siRNA in ICP1 cells for 48 h, as measured by propidium iodide staining for DNA content. (**G**–**I**) Proliferation analysis after transfection of ICP1 cells with a *GSTA2* overexpression plasmid and *GSTA2* siRNA for 48 h, as determined by EdU staining. * *p* < 0.05; ** *p* < 0.01.

To explore the role of *GSTA2*, overexpression and interference experiments were performed in ICP1 cells to evaluate the impact of *GSTA2* on cell proliferation. We assessed the relative expression levels of cell cycle-related genes, including *CDKN2B*, *CCNB2*, *CCND1* and *CCNG2*, by qPCR. *GSTA2* overexpression markedly upregulated the expression of *CCND1*, *CCNB2*, *CDKN2B*, and *CCNG2*, while transfection with si-*GSTA2* significantly downregulated their expression (*p* < 0.05) (Figure 4A,B).

Flow cytometry was used to evaluate the cell cycle status of ICP1 cells. The findings demonstrated that overexpression of *GSTA2* led to a notable decrease in the number of cells in the G0/G1 phase, an increase in the number of cells in the S phase, and a significant increase in cell proliferation activity (*p* < 0.01) (Figure 4C,D). However, *GSTA2* knockdown did not have a significant impact on cell proliferation (Figure 4E,F). Additionally, the results of the EdU assay demonstrated that *GSTA2* overexpression significantly promoted the viability of ICP1 cells, leading to a significant increase in cell proliferation, while *GSTA2* knockdown inhibited the proliferation of ICP1 cells (*p* < 0.01) (Figure 4G–I). These findings suggest that *GSTA2* can promote the proliferation of ICP1 cells.

### 2.11. GSTA2 Promotes the Differentiation of ICP1

**Figure 5:**
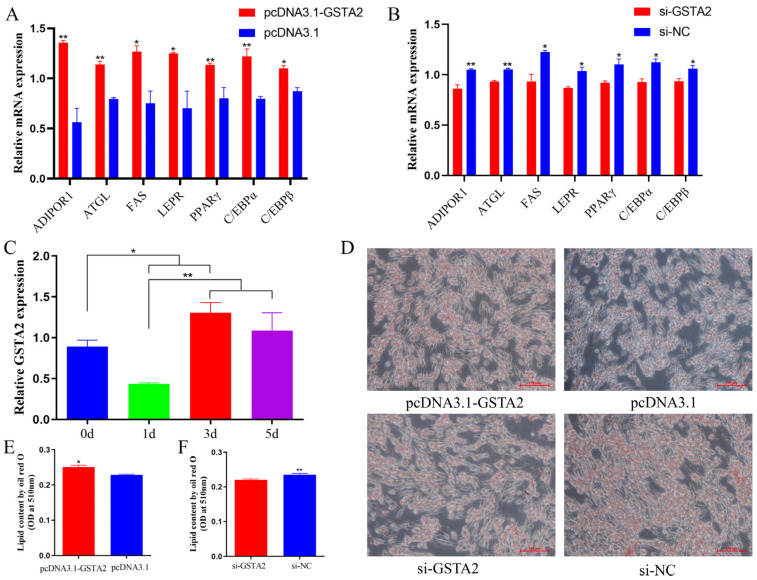
The *GSTA2* gene promotes adipogenesis in chicken preadipocytes. (**A**,**B**) Relative mRNA expression levels of differentiation-related were genes detected by qPCR after transfection of chicken ICP1 cells with a *GSTA2* overexpression plasmid and *GSTA2* siRNA for 48 h. (**C**) The relative expression of GSTA2 in ICP1 cells during a five-day differentiation process was detected by qPCR. (**D**–**F**) Oil Red O staining results and quantification of lipid droplets in chicken ICP1 cells after transfection of a *GSTA2* overexpression plasmid and *GSTA2* siRNA for 48 h. * *p* < 0.05; ** *p* < 0.01.

To further elucidate the potential function of *GSTA2*, the expression levels of adipocyte differentiation-related genes, including *ADIPOR1*, *ATGL*, *FAS*, *C/EBPβ*, *PPARγ*, *C/EBPα* and *LEPR*, were quantified using qPCR after a 3-day period of in vitro induction of chicken preadipocyte differentiation. Compared to the control group, the overexpression of *GSTA2* resulted in significant upregulations of differentiation-related genes, such as *C/EBPα*, *ADIPOR1*, *ATGL*, and *PPARγ* (*p* < 0.01), along with notable increases in the expression levels of *FAS*, *C/EBPβ* and *LEPR* (*p* < 0.05) (Figure 5A). Conversely, interference with the *GSTA2* gene resulted in significant downregulations of *ADIPOR1* and *ATGL* expression (*p* < 0.01), as well as notable decreases in the expression levels of *FAS*, *LEPR*, *C/EBPβ*, *PPARγ*, and *C/EBPα* (*p* < 0.05) (Figure 5B).

Furthermore, the expression pattern of *GSTA2* throughout the differentiation process of ICP1 cells was investigated. The findings revealed a substantial increase in *GSTA2* expression during the late stages of differentiation (3 d and 5 d) compared to the early stage (1 d) of differentiation (*p* < 0.01) (Figure 5C). Oil Red O staining was used to evaluate the lipid droplet content in ICP1 cells following either *GSTA2* overexpression or knockdown. The findings revealed a significant increase in the lipid droplet content in adipocytes upon *GSTA2* overexpression (*p* < 0.01), while the opposite effect was observed when *GSTA2* was knocked down (*p* < 0.05) (Figure 5D–F). These results show that *GSTA2* can promote the differentiation of ICP1 cells.

## 3. Discussion

Artificial selection for commercial broilers and laying hens primarily focuses on traits important for growth or reproduction. During the breeding process, population differentiation occurs, resulting in genetic changes in animals. Continuously selected breeds may have different fixed genotypes as a result. Furthermore, artificial selection plays a role in determining both the quantity and distribution of gene mutations throughout the domestication process [29,30]. Among all populations, the II genotype was the most common, while the DD genotype was the least frequent. It is worth noting that the RW population only had the II genotype, and the *I* allele tended to be fixed in the RW chicken population. Moreover, the RW population showed high or moderate levels of genetic differentiation from the GX, F2, GS, and QY populations, indicating limited gene flow and genetic exchange. These results suggest that different populations may have experienced distinct and sustained selective pressures, with RW potentially experiencing the strongest selection pressure.

To explore the potential effect of the 198-bp SVs in the *GSTA2* promoter region on the F2 resource population, we investigated the correlation between SVs and meat and carcass quality traits in the F2 resource population. The results showed significant correlations between the *GSTA2* SVs and adipose traits such as SFTs, AFWs, and LMCFs. Among all the correlated traits, the II genotype had higher values than the ID genotype, and the DD genotype was not detected in the F2 population. We hypothesize that chickens with the II genotype have greater fat deposition during development. Subcutaneous fat and abdominal fat are typically discarded during chicken meat processing, while intramuscular fat content is an important factor influencing flavor, meat quality, tenderness, and juiciness [31]. As people’s living standards continue to improve, consumer demand for chicken meat has shifted from quantity to quality (emphasizing attributes such as flavor, color, and texture). Therefore, maintaining a balance between intramuscular fat and abdominal fat is of significant economic value for high-quality broiler production. It has been previously shown that *GSTA2* may be involved in the differentiation of adipocytes in mouse 3T3-L1 cells [32], suggesting a potential association between *GSTA2* and fat deposition in chickens.

Maintaining energy balance is crucial for regulating growth and development, and the liver, which is the primary metabolic organ in the body, plays a pivotal role in this process. The liver governs the synthesis and metabolism of proteins, fats, and carbohydrates, as they contribute to overall energy balance [33]. Additionally, the *GSTA2* gene is highly expressed in liver tissue. Therefore, to investigate the reasons behind the variations in fat content among individuals with different genotypes, we selected the liver to examine the expression levels of *GSTA2* in different genotypes. The results revealed that the II genotype had markedly greater expression in the liver than the ID and DD genotypes (Figure 3A). Consistently, individuals with the II genotype exhibited the highest SFTs, AFWs, and LMCFs. Increasing evidence suggests that gene expression regulation is a complex process in eukaryotes involving various factors, such as gene 5′ and 3′ untranslated regions, promoters and introns [34,35]. Moreover, transcription factors are pivotal in governing gene expression. We predicted TFBSs within the inserted fragment of the II genotype, and the results revealed the existence of binding sites for transcription factors such as SP1, C/EBPα, and OCT-1 (Figure S3). The luciferase activity results indicated that the pGL3-II vector exhibited a greater level of transcriptional activity than the pGL3-C vector (Figure 3B). This difference indicates that the inserted fragment in the *GSTA2* promoter region may contain transcription factors that activate gene expression, potentially enhancing the transcriptional activity of the *GSTA2* gene and promoting fat deposition in chickens.

Next, we mutated the binding sites of the three candidate transcription factors, SP1, OCT-1, and C/EBPα, within the inserted fragment. Compared with those in the pGL3-II group, the luciferase activities of the PGL3-SP1, PGL3-C/EBPα, and PGL3-OCT-1 groups were significantly lower, indicating that SP1, C/EBPα, and OCT-1 are potential transcription factors that affect the expression of the *GSTA2* gene. A previous study showed that C/EBPα is an essential transcription factor required for fat generation [36]. We transfected DF-1 cells with *C/EBPα* overexpression vectors and the luciferase vectors PGL3 and PGL3-DD, either individually or in combination. The findings demonstrated notable increases in luciferase activity in the PGL3-DD+pcDNA3.1-C/EBPα group compared to the PGL3-DD and PGL3 control groups (Figure 3D). Additionally, the overexpression of *C/EBPα* in ICP1 cells significantly enhanced the expression of the *GSTA2* gene (Figure 3E). These results strongly suggest that C/EBPα functions as a transcription factor for *GSTA2* and is capable of regulating its gene expression. Similar research has shown that transcription factors bound to a 61-bp SV in the *RIN2* gene affect its transcriptional activity, which may alter chicken fat traits [37]. These findings suggest that changes in *GSTA2* expression may be related to chicken fat deposition and that the transcription factors binding to the 198-bp SV sequence influence *GSTA2* expression.

The results of subcellular localization prediction indicated that *GSTA2* may play a regulatory role in the cytoplasm. According to the results of the protein–protein interactions, GPX2, GPX3, GPX7, and GPX8 are all members of the glutathione peroxidase (GPX) family. GPX is an established antioxidant enzyme responsible for facilitating the reduction in hydrogen peroxide and lipid peroxides. This process is accomplished by utilizing reduced glutathione as a substrate, protecting cells from oxidative damage [38]. Earlier research revealed that the overexpression of *GPX2* in 3T3-L1 cells can inhibit adipocyte proliferation and adipogenic differentiation while increasing lipid degradation. In C2C12 cells, overexpression of *GPX2* promotes myoblast proliferation and myogenic differentiation [39]. Compared to that in human preadipocytes, the expression of the *GPX3* (also known as pGPX) gene is increased in human adipocytes [40]. During the process of adipogenesis in bovine intramuscular preadipocytes, the expression of *GPX3* starts to increase beginning on day 2 and reaches its peak after 4 days of stimulation [38]. In a study on mice, the absence of *GSTO1* was found to enhance the inflammatory response triggered by LPS while mitigating the inflammatory effects of a high-fat diet on glucose tolerance and insulin resistance [41]. These findings further imply a potential connection between the *GSTA2* gene and adipogenesis.

In this study, we observed that the expression levels of the *GSTA2* gene were particularly prominent in liver and leg muscle tissues but were decreased in the heart, spleen, lung, and other tissues. In birds, the liver plays a crucial role in synthesizing fats, whereas adipose tissue serves as a repository for triglycerides [42]. To explore the potential relationship between *GSTA2* and chicken fat traits, we examined the expression of *GSTA2* in abdominal fat from chickens with high and low abdominal fat contents. *GSTA2* exhibited greater expression in the high-fat group than in the low-fat group. Additionally, GSTA2 expression was significantly positively correlated with the abdominal fat percentage in chickens (Figure 2). These results suggest that *GSTA2* may promote fat deposition. Previous studies have shown that carnosic acid treatment of 3T3-L1 cells inhibits lipid absorption and adipocyte differentiation while simultaneously promoting *GSTA2* expression [32]. In the low abdominal fat group of chickens, there was a notable upregulation of *ALDH1A1* gene expression in adipose tissue. When *ALDH1A1* is overexpressed in vitro, it hinders the proliferation and differentiation of chicken preadipocytes. Furthermore, in vivo overexpression of *ALDH1A1* leads to a reduction in fat deposition in chickens. However, the regulatory mechanisms of this gene are completely opposite in mice [43,44]. During the process of fat deposition in chickens, the *GSTA2* gene may play a regulatory role opposite to that of the *ALDH1A1* gene.

For in vitro experiments, we synthesized overexpression vectors and siRNAs for *GSTA2*. A series of experimental results revealed that *GSTA2* overexpression significantly upregulated the expression levels of cell proliferation-related genes, including *CCND1*, *CCNB2*, *CDKN2B*, and *CCNG2*. Moreover, it induced a substantial decrease in the cell population in the G0/G1 phase while concurrently promoting an increase in the cell population in the S phase and enhancing cell proliferation. Furthermore, it significantly promoted the viability of ICP1 cells, resulting in a significant increase in the cell proliferation rate. Conversely, knocking down *GSTA2* expression had the opposite effect. These results indicate that the upregulation of *GSTA2* can promote the proliferation of ICP1 cells.

In differentiated ICP1 cells, overexpression of *GSTA2* significantly upregulated the expression levels of differentiation-associated genes, including *ADIPOR1*, *ATGL*, *PPARγ*, *C/EBPα*, and *C/EBPβ*. It also significantly increased the lipid droplet content in adipocytes, while knocking down *GSTA2* expression had the opposite effect. Multiple factors regulate the process of adipogenesis, with *PPARγ* and *C/EBPα* being the most well-known factors known to promote adipocyte differentiation in vitro [45,46]. Preadipocytes do not express *C/EBPα* or *PPARγ* initially; however, these factors become activated prior to the expression of the majority of adipocyte-related genes. Importantly, numerous genes associated with adipogenesis feature binding sites for *C/EBPα* and *PPARγ* in their promoter regions [47]. Interestingly, the promoter region of the *GSTA2* gene also contains a binding site for C/EBPα. The temporal sequence of gene expression during adipogenesis suggests that *C/EBPβ* and *C/EBPδ* may be expressed prior to *C/EBPα*. These early-expressed factors, C/EBPβ and C/EBPδ, are believed to transmit hormonal signals for adipocyte differentiation to downstream effectors, such as *C/EBPα* and *PPARγ*, thereby promoting adipocyte generation [48,49]. Additionally, the expression of *GSTA2* in ICP1 cells was markedly greater in the later stages of differentiation than in the early stages. Earlier research demonstrated that elevated levels of *FOXO6* in chickens inhibit the proliferation of preadipocytes by inducing G1 cell cycle arrest. This upregulation of *FOXO6* also leads to increased expression of cell cycle markers, such as *CCNG2*. Additionally, *FOXO6* plays a role in regulating early adipogenesis by suppressing the expression of crucial regulators of adipogenesis, including *FABP4*, *PPARγ* and *C/EBPα* [50]. Upregulation of the *FTO* gene in chickens significantly increases the expression of growth-related genes such as *CCND1*, *CCND2*, and *CCNB2*; enhances cell proliferation activity and rate; elevates the expression of *PPARγ*, *C/EBPα*, and *C/EBPβ*; and promotes triglyceride accumulation in ICP1 cells, thereby facilitating the proliferation and differentiation of ICP1-producing cells [51]. In ICP1 cells, the *GSTA2* gene exhibits regulatory functions similar to those of *FTO*, indicating that the upregulation of *GSTA2* can enhance the proliferation and differentiation of ICP1 cells. Nevertheless, additional research is needed to determine the specific molecular mechanisms involved.

## 4. Materials and Methods

### 4.1. Collection of Animal Samples

In total, 1205 chicken DNA samples were collected from nine different populations, including Qingyuan spotted-brown chickens (QY, *n* = 60, male), ISA Brown laying hens (ISA, *n* = 67, female), Guangxi chickens (GX, *n* = 69, male), Wenchang chickens (WC, *n* = 46, male), Gushi chickens (GS, *n* = 50, male), Ningdu chickens (ND, *n* = 71, male), Tianlu N409 chickens (N409, *n* = 428, male), Recessive White Rock chickens (RW, *n* = 55, male), and an F2 population (F2; *n* = 359) (184 males and 175 females). In this study, all chickens were euthanized by intraperitoneal injection of 2% pentobarbital (Beijing Siyuan Technology Co., Ltd., Beijing, China), followed by bleeding through the carotid artery after 2–3 min. The DNA samples used in this study were sourced from a chicken breed resource library that is under the care and maintenance of our laboratory. Among the nine chicken populations, QY, ND, GS, GX, and WC represent Chinese-local chicken breeds, ISA represents commercial layer hen breeds, and RW represents commercial broiler breeds. The F2 population comprises full-sibling hybrid offspring resulting from the crossbreeding of Xinghua (XH) and RW chickens. XH chickens are a Chinese domestic chicken breed known for slow growth, whereas N409 is a hybrid strain derived from GX chickens. The meat quality and carcass traits of all individuals in the F2 population were measured for association analysis. The subcutaneous fat thickness near the tail region of the chicken’s back was measured using a caliper. The intact leg muscle tissue samples were weighed, and visible fat and fascia on the surface of the leg muscle were removed. The tissue was then minced and mixed before being subjected to Soxhlet extraction using petroleum ether to determine the crude fat content of the leg muscle [52]. Detailed information regarding the measurement methods has been previously described [53].

For the analysis of *GSTA2* tissue expression profiles, eleven different tissues were collected from a group of four 20-week-old QY female chickens. These tissues included the spleen, heart, kidney, liver, lung, duodenum, small intestine, breast muscle, ovary, abdominal fat, and leg muscle. Furthermore, we obtained liver tissue samples from twelve 4-week-old XH female chickens to investigate the expression of *GSTA2* across different genotypes.

### 4.2. Bioinformatics Analysis

The amino acid sequences of *GSTA2* from 12 species were analyzed by MEGA 11.0 software. After removing sequences with lower consistency, a phylogenetic tree was constructed using the bootstrap and neighbor-joining methods. The STRING database was used to predict *GSTA2*-interacting proteins in chickens (https://cn.string-db.org/cgi/input?sessionId=bPpsyQ0kzZ8C&input_page_show_search=on, accessed on 1 November 2023). The UniProt database was used to predict the subcellular localization of *GSTA2* (https://www.uniprot.org/, accessed on 6 December 2023). The prediction of transcription factors (TFs) within the 198-bp SVs of *GSTA2* was performed using Alibaba 2.1 (http://gene-regulation.com/pub/programs/alibaba2/index.html, accessed on 7 December 2023).

### 4.3. Genetic Variation and Genotyping

A 198-bp SV (EVA submission no. 729567) within the *GSTA2* gene was identified (designated ATG as +1; this SV is located 3378 bp upstream of the ATG codon) by analyzing previously published resequencing data of XH and RW chickens from our laboratory [54]. To acquire additional insights into the distribution of polymorphisms within the 198-bp SV, we employed PCR and agarose gel electrophoresis techniques to identify the genotypes present in the nine distinct populations. The genotyping primers used for *GSTA2* are listed in Table S1. Each 10 μL PCR mixture contained 5 μL of 2× Taq PCR StarMix (GenStar, Beijing, China), 1.0 μL of template DNA (80 ng/µL), 0.5 μL of each primer (10 µM), and 3.0 μL of water.

The PCR procedure included initial denaturation at 94 °C for 3 min, followed by 34 cycles of denaturation at 94 °C for 30 s, annealing at 59 °C for 30 s, extension at 72 °C for 30 s, and a final extension at 72 °C for 10 min. Subsequently, the PCR products were analyzed through 1.5% gel electrophoresis, and the different genotypes were validated by DNA sequencing (Tsingke, Guangzhou, China).

The genotypic and allelic frequencies of the nine populations were determined directly, and the Hardy–Weinberg equilibrium (HWE) was assessed using the SHEsis website [55]. Additionally, genetic indices, including expected heterozygosity (He), observed heterozygosity (Ho), effective allele number (Ne), and polymorphic information content (PIC), were calculated using PopGene software (version 1.3.1; Edmonton, AB, Canada).

### 4.4. Cell Culture

The DF-1 cell line used in this study was a long-term preserved cell line obtained from our laboratory. The culture medium utilized for these cells contained 10% FBS (Gibco, Bethesda, MD, USA), 89% basal DMEM (Biological Industries, Bet-Haemek, Israel), and 1% penicillin and streptomycin (Invitrogen, Carlsbad, CA, USA). ICP1 cells were generously provided by Northeast Agricultural University (Harbin, China). The culture conditions for the ICP1 cells were the same as those for the DF-1 cells. Primary preadipocytes were isolated from the abdominal fat of 3-week-old chickens. Initially, the fat tissue was carefully dissected from the abdominal cavities of the chickens, and then the fat tissue was minced using scissors. Collagenase I was used to digest the tissue at 37 °C for 30 min, with gentle agitation every 5 min during the digestion period, to obtain a single-cell suspension. Subsequently, the cell suspension was centrifuged at 1300 rpm for 5 min to collect the primary preadipocytes. The culture conditions for the primary preadipocytes were the same as those for the DF-1 cells. The cell cultures were maintained in a CO_2_ incubator at 37 °C with a CO_2_ concentration of 5%.

### 4.5. cDNA Synthesis and Quantitative Real-Time PCR (qPCR)

Total RNA from cells and tissues was extracted using a TRIzol reagent kit (Takara, Dalian, China) according to the manufacturer’s instructions; RNA quality was assessed using gel electrophoresis and spectrophotometry; and cDNA synthesis was performed using a PrimeScript RT reagent kit (Takara, Dalian, China), followed by qPCR analysis. The relative expression levels were calculated using the 2^–ΔΔCt^ method, and statistical significance was evaluated using ANOVA and Duncan’s test. All reactions were conducted with four biological replicates and three technical replicates. β-actin was used as the internal control. The qPCR primers used are listed in Table S1.

### 4.6. Dual-Luciferase Reporter Assay

Dual-luciferase reporter plasmid: The plasmids for the *GSTA2* gene SVs, pGL3-II and pGL3-DD, and the mutated plasmids for the transcription factors, pGL3-SP1, pGL3-C/EBPa, and pGL3-OCT-1, were all synthesized by Gene Create (Wuhan, China). The sequences of the plasmids with different lengths can be found in Table S3. DF-1 cells were utilized to conduct the dual-luciferase reporter assay. Transfection was performed when the cell growth density reached approximately 50–70%. Next, the PGL3-Basic vector plasmid and the internal reference PRL-TK-Renilla vector plasmid were cotransfected into the cells (transfection dosage: reporter gene plasmid: PRL-TK=10:1). After transfection for 48 h, the luciferase activity was measured in accordance with the instructions provided by the manufacturer of the Dual-Glo® Luciferase Assay System (Promega, Madison, WI, USA).

### 4.7. RNA Oligonucleotides, Plasmid Construction and Cell Transfection

Gene overexpression vectors: pcDNA3.1-GSTA2 for *GSTA2* overexpression (NCBI Reference Sequence: NM_001001776.2), pcDNA3.1-C/EBPa for *C/EBPa* overexpression (NCBI Reference Sequence: NM_001031459.2); The *GSTA2* and *C/EBPa* overexpression plasmids were synthesized by Gene Create (Wuhan, China). The siRNAs used to knock down the *GSTA2* gene were synthesized by RiboBio (Guangzhou, China), and the nonspecific duplex si-NC, which was supplied by RiboBio, served as a control in the experiment. Lipofectamine 3000 reagent (Invitrogen) was used for all transient transfections.

### 4.8. Flow Cytometry Assays

The cell cycle distribution of ICP1 cells was analyzed using flow cytometry. Transfection with siRNA or an overexpression plasmid was performed when the cell density reached 70%. After 48 h of transfection, the ICP1 cells were trypsinized, harvested, fixed in 70% ethanol, and stored at 4 °C for a period of 12–24 h. The fixed cells were then stained with propidium/RNase A (50×) staining solution (Beyotime, Shanghai, China). Flow cytometry analysis was conducted using a BD Accuri^TM^ C6 flow cytometer (BD Biosciences, San Jose, CA, USA) after a 30-min staining period at room temperature in the dark.

### 4.9. 5-Ethynyl-2′-deoxyuridine (EdU) Assay

ICP1 cells were seeded in 12-well plates at approximately 70% confluency, followed by transfection with siRNA or overexpression plasmid. After 48 h, the cells were fixed and stained using a C10310 EdU Apollo in vitro imaging kit (RiboBio, Guangzhou, China). Images of three randomly selected fields were acquired using a fluorescence microscope (Leica DMi8, Wetzlar, Germany), and the number of EdU-stained cells was quantified using ImageJ software (National Institutes of Health, Bethesda, MD, USA).

### 4.10. Oil Red O Staining

When the density of the ICP1 cells reached approximately 70%, the cells were transfected with the overexpression vectors and interfering fragments. After 6 h, a medium containing 0.2% oleic acid was used to replace the cell culture medium. After a 48-h incubation, the cells were fixed with 4% formaldehyde for 30 min and washed with 60% isopropanol. The sections were then stained with Oil Red O reagent for 30 min and imaged using a fluorescence-inverted microscope (TE2000-U; Nikon, Tokyo, Japan). After examination, the Oil Red O dye was extracted with isopropanol, and the absorbance was measured at 510 nm using a Multiskan™ FC (Thermo Scientific, Waltham, MA, USA).

### 4.11. Statistical Analysis

Linear mixed models were employed for the association analysis of the F2 population. Model I (Y_ijkl_ = µ + G_i_ + S_j_ + H_k_ + f_l_ + e_ijkl_) was utilized for meat quality traits, while Model II (Y_ijkl_ = l + G_i_ + S_j_ + H_k_ + f_l_ + b (W_ijkl_ − W(—)) + e_ijkl_) was utilized for carcass traits, with carcass weight included as a covariate in Model II. In these two models, Y_ijkl_ is the observed value, G_i_ represents the fixed effect of genotype, S_j_ is the fixed effect of sex, H_k_ represents the fixed effect of hatch, f_l_ is the fixed effect of family, and e_ijkl_ represents the random error. Detailed information about the mixed model has been described in our previous article [26,33]. All the data in this study were analyzed using SPSS 26.0 (IBM, Armonk, NY, USA). A one-way analysis of variance (ANOVA) followed by post hoc testing was used to compare multiple groups of data. Student’s *t*-tests were used to compare two-group data (* *p* < 0.05, ** *p* < 0.01).

## 5. Conclusions

**Figure 6:**
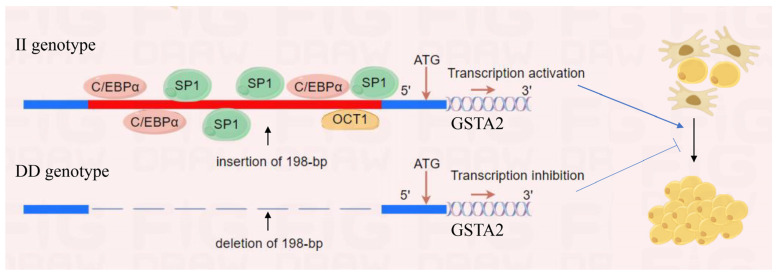
Schematic diagram illustrating the regulatory mechanism of the 198-bp SV in the *GSTA2* gene during chicken adipogenesis (by Figdraw). The 198-bp SV in the *GSTA2* gene promoter affects the binding of multiple transcription factors. These transcription factors regulate the expression of *GSTA2*, thereby promoting the proliferation and differentiation of chicken preadipocytes.

In summary, the 198-bp SVs in the promoter region of the *GSTA2* gene significantly affect fat traits in chickens, with the II genotype being the dominant genotype that promotes fat deposition in the F2 population. An insertion fragment of 198-bp in the II genotype enhances promoter activity and promotes *GSTA2* expression. Furthermore, we observed a significant positive correlation between the expression of *GSTA2* in abdominal fat and the percentage of abdominal fat. Subsequent investigations revealed that *GSTA2* can stimulate the proliferation and differentiation of preadipocytes. Mechanistically, the 198-bp fragment contains binding sites for transcription factors such as C/EBPα, which regulate the gene expression of *GSTA2*, thus affecting adipogenesis, as shown in Figure 6. Our work provides strong evidence for the use of the 198-bp SV in the *GSTA2* promoter region as a molecular marker for molecular breeding in poultry and offers new insights into the key role of the *GSTA2* gene in adipogenesis.

## Figures and Tables

**Table 1:** Genotypic and allelic frequencies and related genetic parameters of the *GSTA2* gene.

Breeds/*n*	Genotypic and Allelic Frequencies					Ho	He	Ne	PIC	*p*-Value (HWE)
	DD	ID	II	D	I					
F2/359	0.00	0.18	0.82	0.09	0.91	0.18	0.17	1.20	0.15	0.062
N409/428	0.11	0.35	0.54	0.29	0.71	0.35	0.41	1.69	0.32	0.004
ND/71	0.00	0.04	0.96	0.02	0.98	0.04	0.04	1.04	0.04	0.856
GS/50	0.00	0.26	0.74	0.13	0.87	0.26	0.23	1.29	0.20	1.00
GX/69	0.19	0.32	0.49	0.35	0.65	0.32	0.45	1.83	0.35	0.014
WC/46	0.00	0.17	0.83	0.09	0.91	0.17	0.16	1.19	0.15	0.518
QY/60	0.05	0.28	0.67	0.19	0.81	0.28	0.31	1.45	0.26	0.507
ISA/67	0.01	0.15	0.84	0.09	0.91	0.15	0.16	1.19	0.15	0.488
RW/55	0.00	0.00	1.00	0.00	1.00	0.00	0.00	1.00	0.00	NA

Note: Ho, observed heterozygosity; He, expected heterozygosity; Ne, effective allele numbers; PIC, polymorphism information content; *p*-value (HWE), *p*-value of Hardy–Weinberg equilibrium; NA indicates not applicable.

**Table 2:** Association analysis of the *GSTA2* 198-bp SV with carcass and meat traits in the F2 population.

Traits	Mean ± SEM		*p*-Value
	II	ID	
SFT (mm)	4.183 ± 0.068 ^a^	3.814 ± 0.146 ^b^	0.022
AFW (g)	28.98 ± 0.983 ^a^	23.684 ± 2.151 ^b^	0.025
LMCF (%)	3.662 ± 0.221 ^a^	3.108 ± 0.265 ^b^	0.001

Note: SEM, standard error of the mean; SFT, subcutaneous fat thickness; AFW, abdominal fat weight; LMCF, content of chicken leg muscle crude fat. Means with different superscripts indicate significant differences (different lowercase letters indicate *p* < 0.05; the same letters indicate no difference, *p* > 0.05).

## Data Availability

All data in the current study can be obtained from the corresponding author upon reasonable request.

## Supplementary Materials

The following supporting information can be downloaded at: https://www.mdpi.com/article/10.3390/ijms25137155/s1.
