# Supplementary material for: Regulatory Effects of 198-bp Structural Variants in the GSTA2 Promoter Region on Adipogenesis in Chickens
Source: Int J Mol Sci. 2024 Jun 28;25(13):7155. doi: 10.3390/ijms25137155 (PMC11241197; doi:10.3390/ijms25137155)
Supplement: Supplementary file 1 [file ijms-25-07155-s001.zip › ijms-3033300-supplementary.pdf]

Supplementary Materials

Regulatory Effects of 198-bp Structural Variants in the *GSTA2* Promoter Region on Adipogenesis in Chickens

Wangyu Li[1,2,3†], Meng Xu[4†], Zihao Zhang[5], Jiaying Liang[1,2,3], Rong Fu[1,2,3], Wujian Lin[1,2,3], Wen Luo[1,2,3], Xiquan Zhang[1,2,3]* and Tuanhui Ren[1,2,3,4]*

1 Department of Animal Genetics, Breeding and Reproduction, College of Animal Science, South China Agri-cultural University, Guangzhou, 510642, China;

2 Guangdong Key Laboratory of Genome and Molecular Breeding of Agricultural Animals and Key Laborato-ry of Chicken Genetic Breeding and Reproduction, Ministry of Agriculture, Guangzhou 510642, China;

3 State Key Laboratory of Swine and Poultry Breeding Industry, Guangzhou, 510642, China;

4 College of Veterinary Medicine, Jilin University, Changchun, 130062, China;

5 College of Coastal Agricultural Sciences, Guangdong Ocean University, Zhanjiang 524088, China;

* Correspondence: xqzhang@scau.edu.cn; tuanhuiren@jlu.edu.cn

† These authors have contributed equally to this work.

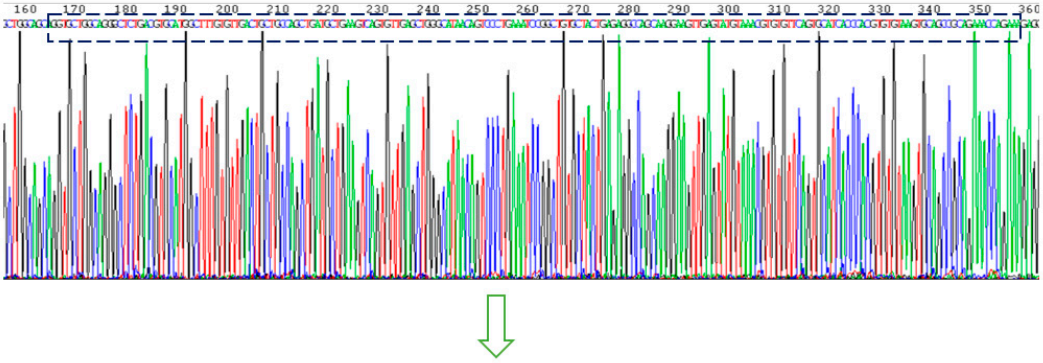

AGGTGCTGGCAGGGCTCTGACGTGCATGGCTTTGTGTTGACTGCTGCCAGCTGAT
GCTGAAGTCAGTGTTGAGCTGGGCATAACAGTCCCTGAAATCCGGCTGTGCTACT
GAGAGGCCAGCAAGGAAGTTGAGTATGTAAACGTGTGTTCAGTGCATCACCCAC
GTGTGTAAGTGCAGCCGCAGAAACCAGAAAGAGGTG

Figure S1. Insertion sequence of the II genotype. The sequence of the inserted fragment of the II genotype in the *GSTA2* gene is 200-bp in length. Interestingly, compared to the II genotype, the DD genotype is missing only the 198-bp segment and has an additional 2-bp (bases CA). However, unfortunately, our current technological means cannot determine whether the CA bases in the DD genotype are a replacement for the II genotype insertion fragment or if the DD genotype is missing the 200-bp segment and retains the CA bases. Therefore, we named this mutation a 198-bp SV. The CA bases in the 200-bp insertion sequence are highlighted in red.

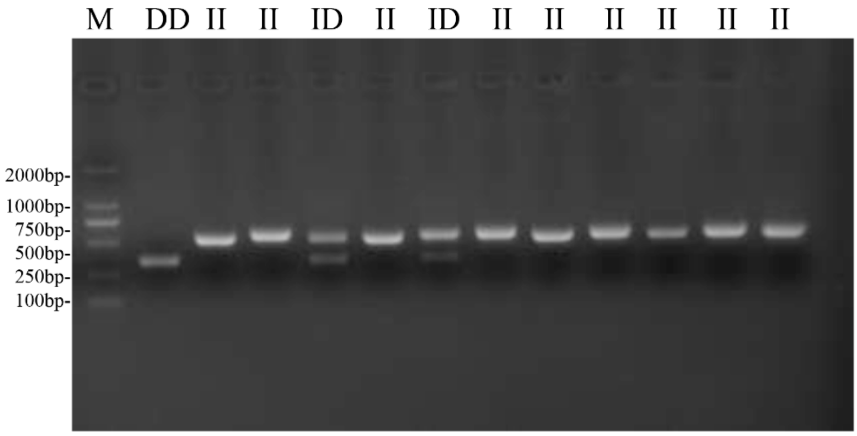

Figure S2. Agarose gel electrophoresis (1.5%) pattern for the *GSTA2* SVs polymorphism. DD (344-bp), ID (542 and 344-bp) and II (542-bp) are the three different genotypes, and M represents DL2000.

```
==================================================================================
seq(    0..   59)     aggtgctggcagggctctgacgtgcatggctttgtgttgactgctgccagctgatgctga
Segments:
1.1.3.0    12   21                =C/EBPalp=
1.1.2.0    13   22                 ====CREB==
0.5.1.0    14   23                  =E1A 12S =
1.1.1.6    14   23                  ==CRE-BP1=
2.1.2.1    14   23                  =RAR-alph=
2.3.3.0    14   23                  =CPE bind=
1.3.1.2    15   24                   ====USF===
9.9.539    38   47                                     ====NF-1==
1.2.1.0    46   55                                             =====E1===
1.2.2.0    46   55                                             ===Myf-3==
1.1.3.0    48   57                                               =C/EBPalp=
9.9.29     48   57                                               ====AP-1==
1.1.1.6    55   64                                                      =ATF-
2.1.1.4    59   68                                                          =
==================================================================================
seq(   60..  119)     agtcagtgttgagctgggcataacagtccctgaaatccggctgtgctactgagaggccag
Segments:
1.1.1.6    55   64    3del=
2.1.1.4    59   68    ====ER===
2.4.1.0    64   73        ====p40x==
2.3.1.0    69   78             ====Sp1===
1.2.8.0    81   90                         ===Olf-1==
2.3.1.0    96   105                                   ====Sp1===
2.3.1.0    111  120                                                  ====Sp1==
==================================================================================
seq(  120..  179)     caaggaagttgagtatgtaaacgtgtgttcagtgcatcacccacgtgtgtaagtgcagcc
Segments:
2.3.1.0    111  120   =
1.1.3.0    124  133       =C/EBPalp=
3.1.2.2    131  140              ===Oct-1==
1.3.1.2    159  168                                       ====USF===
3.5.1.2    174  183                                                      ===Adf
==================================================================================
seq(  180..  239)     gcagaaaccagaaagaggtg
Segments:
3.5.1.2    174  183   -1==
3.5.2.0    191  200              ===Elk-1==
2.3.1.0    192  201               ====Sp1===
```

Figure S3. The predicted results of transcription factor-binding sites in the insertion sequence of the II genotype. The parameters employed in the Alibaba 2.1 online tool are as follows: cons = 75% and classification level K = 4.

Table S1. Pairwise fixation indices (Fsts) of the GSTA2 gene in different chickens.

Breeds	F2	N409	ND	GS	GX	WC	QY	ISA
N409	0.059							
ND	0.009	0.046						
GS	0.002	0.011	0.047					
GX	0.078	0.002	0.179	0.061				
WC	0.000	0.018	0.023	0.005	0.089			
QY	0.013	0.005	0.081	0.007	0.03	0.022		
ISA	0.000	0.023	0.023	0.004	0.097	0.000	0.022	
RW	0.013	0.043	0.009	0.073	0.191	0.049	0.102	0.042

Table S2. Details of the primer pairs used in this study.

Gene name	Primer sequences (5'-3')	Annealing temperature (°C)
GSTA2	F: ATTGCCTTAATCACTGAAC<br>R: TGCCAACAAGATAATCCT	60
CCNB2	F: CAGTAAAGGCTACGAAAG<br>R: ACATCCATAGGGACAGG	58
CDKN1B	GCTGTGCTGGGCTGAA<br>GGACGAAAGGATGTGGG	58
CCNG2	F: TGCCAACAATACCAGAGG<br>R: TACAGAATACCACAATCCC	58
CCND2	F: AACTTGCTCTACGACGACC<br>R: TTCACAGACCTCCAACATC	58
PPARγ	F: TCCTTCCCGCTGACCAAA<br>R: TCCTGCACTGCCTCCACA	60
C/EBPα	F: GACAAGAACAGCAACGAGTACCGC<br>R: CCTGAAGATGCCCCGCAGAGT	60
C/EBPβ	F: GCGGACTGTTTGGCTGCTCT<br>R: CGGGTGAGGCTGATGTAGGTGT	60
ADIPOR1	F: GACAAGAACAGCAACGAGTACCGC<br>R: CCTGAAGATGCCCCGCAGAGT	60
FABP4	F: ATGTGCGACCAGTTTGTG<br>R: TTTGCCATCCCACTTCTG	60
FAS	F: CGCAGGCATAGCAGGAAA<br>R: CCAAAGAAGGAGGCATCAA	60
LEPR	F: CCAACCCTTCCTTGCTAA<br>R: GCCTTCAACCCAACATTC	60
ATGL	F: TGTCCAAAGAAGCACGAAA<br>R: GAGGTATCAGCCCACAGTAGA	60
β-actin	F: CAGGATGCAGAAGGAGATC<br>R: CTGGAAGGTGGACAGGGAG	60
GSTA2-Genotyping	F: GCTTCAACTTAGATGTTCCTGCT<br>R: CAGCGTCACCGTGCTATCAA	60

Note: The primers used for *GSTA2* genotyping were used for SV genotyping.

Table S3. The sequence information of the dual-luciferase reporter plasmids used in this study.

Plasmid name	S
PGL3-II	AGCTGTTACAGTTGGAGGAAGGAGGACTCTGGCTGGCAGCAGGTGCTGGCAGGGCTCTGACGT<br>GCATGGCTTTGTGTTGACTGCTGCCAGCTGATGCTGAAGTCAGTGTTGAGCTGGGCATAACAGT<br>CCCTGAAATCCGGCTGTGCTACTGAGAGGCCAGCAAGGAAGTTGAGTATGTAAACGTGTGTTC<br>AGTGCATCACCCACGTGTGTAAGTGCAGCCGCAGAAACCAGAAAGAGGTGGATGTTGCAGCA<br>CTCCCAGCACGAAGCTGGGCCACCTCCA
PGL3-DD	AGCTGTTACAGTTGGAGGAAGGAGGACTCTGGCTGGCAGCCAGATGTTGCAGCACTCCCAGCA<br>CGAAGCTGGGCCACCTCCA
PGL3-SP1	AGCTGTTACAGTTGGAGGAAGGAGGACTCTGGCTGGCAGCAGGTGCTGGCAGGGCTCTGACGT<br>GCATGGCTTTGTGTTGACTGCTGCCAGCTGATGCTGAAGTCAGTGTATAACAGTCCCTGAAATT<br>ACTGAAGGAAGTTGAGTATGTAAACGTGTGTTCAGTGCATCACCCACGTGTGTAAGTGCAGCC<br>GCAGAAACCAGGATGTTGCAGCACTCCCAGCACGAAGCTGGGCCACCTCCA
PGL3-<br>C/EBPa	AGCTGTTACAGTTGGAGGAAGGAGGACTCTGGCTGGCAGCAGGTGCTGGCAGTGCATGGCTTT<br>GTGTTGACTGCTGCCGAAGTCAGTGTTGAGCTGGGCATAACAGTCCCTGAAATCCGGCTGTGC<br>TACTGAGAGGCCAGCAAGATGTAAACGTGTGTTCAGTGCATCACCCACGTGTGTAAGTGCAGC<br>CGCAGAAACCAGAAAGAGGTGGATGTTGCAGCACTCCCAGCACGAAGCTGGGCCACCTCCA
PGL3-OCT-1	AGCTGTTACAGTTGGAGGAAGGAGGACTCTGGCTGGCAGCAGGTGCTGGCAGGGCTCTGACGT<br>GCATGGCTTTGTGTTGACTGCTGCCAGCTGATGCTGAAGTCAGTGTTGAGCTGGGCATAACAGT<br>CCCTGAAATCCGGCTGTGCTACTGAGAGGCCAGCAAGGAAGTTGCGTGTGTTCAGTGCATCACCCACGTC
